# A Preliminary Study on Contrast Enhanced Ultrasound Characteristics of Solid Pseudopapillary Neoplasms and Pancreatoblastoma in Children

**DOI:** 10.3390/diagnostics16030474

**Published:** 2026-02-03

**Authors:** Yuxin Tang, Juan Wang, Lirong Zhu, Jingyu Chen, Hongli Zhai, Yi Tang

**Affiliations:** 1 Department of Ultrasound, Children’s Hospital of Chongqing Medical University, National Clinical Research Center for Child and Adolescents’ Health and Diseases, Ministry of Education Key Laboratory of Child Development and Disorders, Chongqing Municipal Health Commission Key Laboratory of Chidren’s Vital Organ Development and Diseases, Chongqing 400010, China; 2 Department of Ultrasound Medicine, Renji Hospital, School of Medicine, Chongqing University, Chongqing 400062, China

**Keywords:** solid pseudopapillary neoplasms, pancreatoblastoma, children, contrast enhanced ultrasound

## Abstract

**Objective:** Solid pseudopapillary neoplasms (SPN) and pancreatoblastoma (PB) have a low overall incidence but represent the most common pancreatic tumors in childhood. Currently, there is a lack of systematic descriptions of contrast-enhanced ultrasound (CEUS) features for these two tumors in pediatric populations. This study aims to retrospectively analyze and compare the CEUS characteristics of SPN and PB to explore key imaging differentiation points. **Methods:** This retrospective study collected data on 22 patients with solid pseudopapillary neoplasms and pancreatic blastomas of the pancreas who were pathologically diagnosed at a children’s hospital between September 2019 and May 2025. The ultrasound contrast-enhanced imaging findings for both tumor types were summarized and analyzed. Two physicians with different levels of experience performed qualitative analysis of the contrast-enhanced images, while quantitative analysis was conducted using time–intensity curve (TIC) analysis software. **Results:** This study included a total of 22 pediatric patients (19 with SPN and 3 with PB). Significant differences existed between the two groups in age (13.51 years vs. 2.94 years) and Ki-67 index (5.00% vs. 30.00%). Qualitative analysis revealed high heterogeneity in SPN enhancement patterns, with capsular enhancement with cystic components being the most common (42.11%). All PBs (100%) consistently demonstrated the “disorganized nourishing vessels” sign. Quantitative analysis revealed that PBs exhibited numerically higher IMAX values (179.84% vs. 60.56%) and faster WoR trends (773.88 vs. 275.38). Inter-observer consistency analysis supported measurement reliability (key parameters ICC > 0.80). **Conclusions:** This preliminary study indicates differences in CEUS characteristics between pediatric SPN and PB; PB tends to exhibit rapid, high enhancement with chaotic feeding vessels and rapid washout, whereas SPN more commonly presents with moderate, slow enhancement patterns, often accompanied by features associated with cystic components. These findings provide new hemodynamic clues for their imaging differentiation. Given the extremely small sample size of PB cases, the above conclusions should be regarded as preliminary hypotheses awaiting validation in future large-scale studies.

## 1. Introduction

Malignant pancreatic tumors are extremely rare in the pediatric population (0.018 per 100,000) [1]. Biological behavior and prognosis vary significantly among different tumor types, making accurate preoperative diagnosis crucial for developing individualized treatment plans. The most common pancreatic tumor in children is the solid pseudopapillary neoplasm (SPN) [2]. In 1996, the WHO defined it as a solid pseudopapillary tumor [3]. SPN commonly affects young females, presenting clinically with abdominal discomfort, bloating, or incidentally discovered abdominal masses. As a low-grade malignant tumor, it exhibits favorable biological characteristics and a less aggressive clinical course, typically growing slowly. Complete surgical resection generally yields a favorable prognosis: Ayiguzaili et al. [4] followed 18 pediatric SPN patients who underwent surgery without chemotherapy or radiotherapy, reporting no recurrence or metastasis at 6-month to 2-year follow-up. PB commonly occurs within the first decade of life, accounting for nearly 25% of all pancreatic lesions in children aged 1–5 years [2,5], with a slightly higher incidence in males. Clinical manifestations are often nonspecific, presenting as intermittent abdominal pain accompanied by nausea, vomiting, and loss of appetite. Treatment relies on a multidisciplinary approach centered on surgical resection supplemented by neoadjuvant chemotherapy. Complete surgical resection is a key prognostic factor; patients with unresectable tumors have survival rates below 5 years [6,7,8]. Chemotherapy provides surgical opportunities for most unresectable or metastatic cases. Therefore, accurate diagnosis and differential diagnosis are crucial for formulating and adjusting treatment plans.

Conventional ultrasound is the preferred diagnostic method for pediatric pancreatic diseases. However, due to the pancreas’s deep location, image quality is easily affected by factors such as patient positioning, intestinal gas, and body fat percentage. Furthermore, conventional color Doppler ultrasound struggles to accurately depict tumor perfusion patterns and fails to provide detailed hemodynamic information, limiting its ability to assess tumor malignancy. Additionally, conventional ultrasound lacks quantitative parameters, making results highly operator-dependent and subject to significant subjective interpretation. Contrast-enhanced ultrasound (CEUS) utilizes ultrasound microbubble contrast agents based on acoustic impedance mismatch with body tissues, delivering clearer images of lesions or tissue microcirculatory perfusions. By constructing time–intensity curves (TICs), it delivers quantitative parameters that objectively reflect hemodynamic information of lesions or tissues, thereby enhancing the specificity, sensitivity, and objectivity of ultrasound examinations [9,10]. CEUS is increasingly applied in pediatric radiology practice [11]. However, due to the rarity of pancreatic masses in children, the role of CEUS in evaluating pediatric SPN and PB remains understudied.

This study aims to investigate differences between SPN and PB in CEUS dynamic enhancement patterns and quantitative parameters, with the goal of improving preoperative diagnostic accuracy for these two tumors and providing valuable imaging-based differential diagnostic insights.

## 2. Materials and Methods

### 2.1. Participant

**Figure 1:**
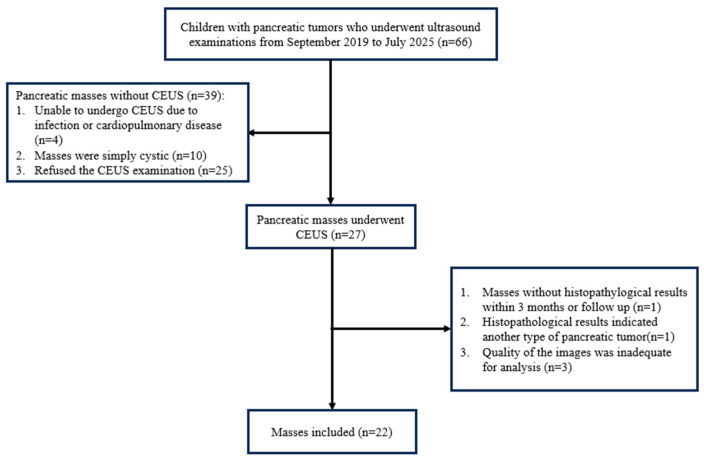
Research flowchart.

A retrospective analysis was conducted on the clinical data of 66 pediatric patients with pancreatic masses detected by abdominal ultrasound examinations performed at the Department of Ultrasound, Chongqing Medical University Children’s Hospital, from September 2019 to May 2025. Inclusion criteria: (1) Age < 18 years; and (2) Ultrasound detection of a pancreatic mass. Exclusion criteria: (1) Inability to undergo CEUS due to cardiopulmonary disease or drug allergy; (2) Lesion being purely cystic without solid component; (3) Lack of definitive histopathological results within 3 months or during follow-up; (4) Histopathological findings indicative of other pancreatic tumor types; and (5) Insufficient image quality after software correction to complete TIC analysis and quantitative parameter assessment. The final diagnosis for all cases was confirmed by histopathological examination of specimens obtained through surgical resection. Ultimately, 22 patients were included in the study (Figure 1).

### 2.2. Ultrasound Examination and Contrast Agent

Contrast-enhanced ultrasound was performed using an Acuson Sequoia ultrasound system (Siemens Medical Solutions USA, Mountain View, CA, USA) equipped with a 1.0–4.0 MHz 5C1 convex array transducer and integrated SonoLiver software (Image-Arena Version 4.3, TomTec Imaging Systems, Unterschleißheim, Germany). The contrast agent used was SonoVue (sulfur hexafluoride microbubbles, Bracco, Milan, Italy). The microbubbles had an average diameter of 1.5–2.5 μm, comparable to circulating red blood cells. Approximately 20 min post-injection, these microbubbles were exhaled from the lungs via respiration. The administered dose was based on the FDA-recommended pediatric liver imaging dose of 0.03 mL/kg. If the weight-based dose did not achieve optimal contrast enhancement, the dose was adjusted accordingly, with a maximum injection volume not exceeding 2.4 mL per session.

### 2.3. Contrast-Enhanced Ultrasound Imaging

First, perform a conventional ultrasound assessment of pancreatic lesions. Subsequently, switch the ultrasound mode to dual-screen mode and position the target lesion centrally on the screen. The contrast agent dose strictly adheres to the standardized pediatric abdominal CEUS protocol established by Davis et al. [12] (0.03 mL/kg). Inject the contrast agent into the antecubital vein into a calculated volume (≤2.4 mL), followed by a 5 mL saline flush. Timing commenced immediately after injection, dividing the entire imaging process into the arterial phase (10–30 s) and the venous phase (30 s–120 s, up to 4–5 min) [12]. All examinations were performed by sonographers with over 10 years of abdominal ultrasound experience. CEUS data were recorded for 3–6 min and digitally stored on hard drives.

### 2.4. Qualitative Analysis

**Figure 2:**
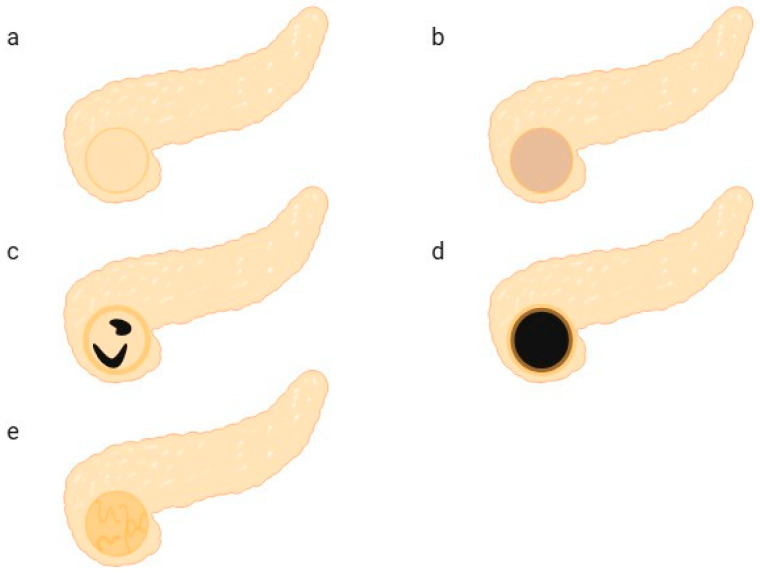
Schematic diagram of qualitative enhancement patterns. (**a**) Pattern I: Homogeneous isointense enhancement. (**b**) Pattern II: Homogeneous hypointense enhancement without cystic components (low vascular lesions with heterogeneous enhancement but no cystic components). (**c**) Pattern III: Capsular enhancement with cystic components. (**d**) Pattern IV: Ring-like enhancement. (**e**) Pattern V: Presence of chaotic, coarse feeding vessels internally.

Two radiologists with over four years of experience in contrast-enhanced ultrasound analyzed the qualitative ultrasound features of the pancreas. In cases of disagreement, a third radiologist with over six years of contrast-enhanced ultrasound experience reviewed the findings to determine the final classification. Based on prior experience and relevant studies, the research team categorized five peak enhancement patterns as shown in Figure 2 [13,14,15]. Pattern I: Homogeneous isointense enhancement (Figure 2a). Pattern II: Homogeneous hyperechoic without cystic components (hypovascular lesion with heterogeneous enhancement but no cystic components) (Figure 2b). Pattern III: Capsular enhancement with cystic components (Figure 2c). Pattern IV: Ring-like enhancement (Figure 2d). Pattern V: Internal presence of chaotic, coarse feeding vessels (Figure 2e).

### 2.5. Quantitative Analysis

**Figure 3:**
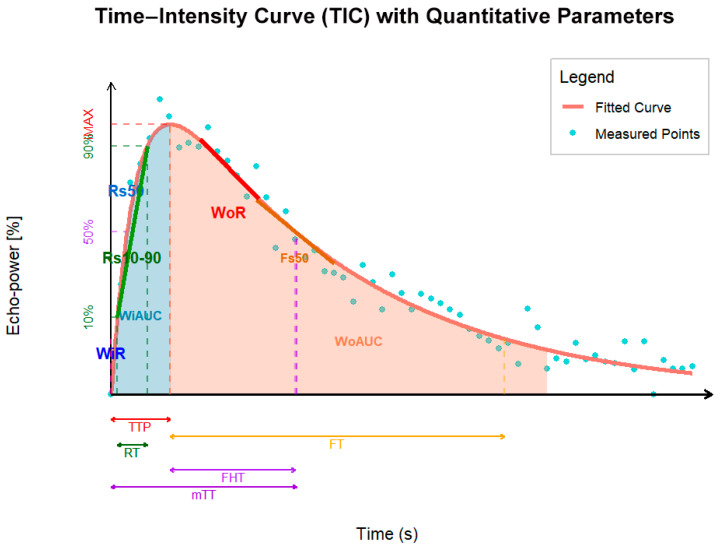
Schematic diagram of quantitative parameters in contrast-enhanced ultrasound.

Quantitative analysis was performed by two physicians with over three years of pediatric ultrasound contrast experience. Regions of interest (ROIs) were manually delineated on dynamic images using Sonoliver software to obtain time–intensity curves (TICs) and quantitative parameters. Place the ROI within the most intensely enhanced solid region of the lesion, avoiding areas with grossly visible cystic changes, necrosis, and calcifications. Place an ROI of identical size within normal tissue at the same depth to minimize variability. Respiratory artifact effects were minimized using compensation techniques. Relative quantitative parameters were derived by calculating ratios of lesion parameters to those of surrounding normal pancreatic parenchyma to mitigate the impact of lesions at varying depths [13]. The following 14 relative quantitative parameters were ultimately obtained: relative peak intensity (rImax), relative time to peak (rTTP), relative rise time (rRT), relative 50% rise slope (rRs50), relative 10–90% rise slope (rRs10–90), relative fall time (rFT), relative 50% fall slope (rFs50), Relative Half-Life Decay Time (rFHT), Relative Mean Transit Time (rmTT), Relative Area Under the Curve (rAUC), Relative Perfusion Area Under the Curve (rWiAUC), Relative Washout Area Under the Curve (rWoAUC), Relative Wash-In Rate (rWiR), and Relative Wash-Out Rate (rWoR) (Figure 3 and Table 1). To maintain consistency in results, measurements taken by Physician A were randomly selected for all subsequent descriptive statistical analyses. Concurrently, we calculated the intraclass correlation coefficient (ICC) between Physician A’s and Physician B’s measurements to assess inter-observer consistency.

### 2.6. Statistical Analysis

Statistical analysis for this study was performed using R software (version 4.5.0; R Foundation for Statistical Computing). Count data are expressed as case numbers (percentages). For data not following a normal distribution, median values (M, [P25, P75]/M, [Min, Max]) were used. We calculated the weighted Kappa coefficient between the independent initial assessments of two physicians to evaluate agreement in enhanced pattern classification. A Kappa value > 0.60 is considered to indicate substantial agreement. Intraclass correlation coefficients (ICCs) were used to assess inter-observer or intra-observer consistency in quantitative measurements. The “two-way mixed” model with “absolute agreement” type was selected for ICC calculation. An ICC value > 0.75 is considered to indicate good agreement.

### 2.7. Ethical Statement

This study was approved by the Ethics Committee of Chongqing Children’s Hospital, Chongqing Medical University, in accordance with the World Medical Association’s Declaration of Helsinki (Approval No. 358/2024 Ethics Committee [Research], approval date: 10 October 2024). Informed consent was obtained from the parents or legal guardians of 22 children.

## 3. Results

### 3.1. Baseline Characteristics

The study included 22 pancreatic tumors, comprising 19 solid pseudopapillary neoplasms and 3 pancreatic blastomas. Baseline clinical characteristics, including age, sex, maximum tumor diameter, and Ki-67 index, are summarized in Table 2.

### 3.2. Qualitative Analysis of CEUS

Two sonographers independently assessed the CEUS dynamic images, demonstrating good inter-observer agreement (weighted Kappa = 0.70). The assessment results are shown in Table 3. The SPN group (*n* = 19) exhibited considerable heterogeneity in enhancement patterns. The most common pattern was Pattern III, observed in eight cases (42.11%), reflecting the pathological characteristics of SPN frequently associated with hemorrhage and cystic changes. Pattern II was the second most frequent, seen in five cases (26.32%). Additionally, four cases (21.05%) demonstrated pattern IV, two cases (10.53%) showed pattern I, and pattern V was not observed.

Three PB cases demonstrated highly consistent qualitative and quantitative analysis. In the early enhancement phase, all three (100%, 3/3) PB lesions exhibited rapid perfusion and heterogeneous hyperenhancement. Chaotic, tortuous feeding vessels were visible within the tumor, followed by rapid diffuse hyperenhancement. The heterogeneous enhancement exhibited a “honeycomb” appearance, suggesting partial necrosis or hemorrhage within the lesion. In the late phase, all cases (100%, 3/3) demonstrated rapid contrast agent washout with markedly reduced enhancement, shifting from early hyperenhancement to isointense or hypointense signals.

Due to the small sample size of only three cases in the PB group, this study did not perform any statistical comparisons or significance tests on the distribution of qualitative characteristics between the two groups. Pattern V was concentrated in the PB group and absent in the SPN group. This finding represents only a preliminary observation warranting attention. Its sensitivity and specificity as a diagnostic discriminative feature must be validated in future large-sample studies. It is important to emphasize that the above description of enhanced pattern distribution in both groups is solely an observational summary based on existing cases.

### 3.3. Quantitative Analysis of CEUS

Descriptive data for CEUS quantitative parameters in the two tumor groups are presented in Table 4. It should be explicitly noted that due to the small sample size of only three cases in the PB group, no statistical comparisons were performed between the two groups in this study. All content in the table below is descriptive in nature. Prior to reporting descriptive quantitative results, we first assessed the measurement reliability. Inter-observer agreement analysis (detailed in Table S1) demonstrated good to excellent consistency for parameters directly related to core tumor hemodynamic characteristics. This indicates that the study’s description of primary perfusion differences between SPN and PB is based on reproducible measurements. Notably, a few composite calculated parameters, such as mTT (ICC = 0.44), exhibited lower consistency. This may stem from their calculation methods being more sensitive to minor variations in ROI placement and curve tailing. Nevertheless, these parameters were not central to the core observational conclusions drawn in this study.

The parameters for the SPN group exhibit a relatively concentrated trend. For instance, the median IMAX reflecting tumor perfusion intensity is 60.56%, with its interquartile range indicating that most cases cluster within the 36.82% to 85.75% range. Regarding blood flow perfusion, the median TTP and RT values are 16.01 s and 15.58 s, respectively, suggesting relatively consistent enhancement rates among tumors in this group. Rs50 and Rs10–90 medians were 8.58 and 6.61, respectively, collectively illustrating the relatively slow contrast agent filling rate in SPN. For contrast agent washout, the median WoR was 275.38, and the median Fs50 was −0.49, indicating a similarly slow washout process.

The PB group (*n* = 3) exhibited markedly distinct parameter values and substantial internal variability. The most striking observation was that its median IIMAX (179.84%) was approximately three times that of the SPN group, suggesting a potentially exceptionally rich blood supply in the PB. Correspondingly, the median values of the rise slopes reflecting perfusion rate (Rs50, Rs10–90) were also substantially higher than those in the SPN group. The median WoR (773.88) in this group was approximately 2.8 times that of the SPN group (275.38). Combined with its significantly higher IMAX and Rs values, this numerical pattern may suggest a “rapid filling–rapid washout” hemodynamic trend in PB tumors. This pattern aligns with the pathological basis of PB as a highly vascularized malignancy, characterized by incomplete tumor vascular endothelium and potential arteriovenous shunting (as documented in the histopathology literature). However, the PB group exhibited high heterogeneity in temporal parameters—suggesting that the mere three PB cases may not represent a homogeneous hemodynamic pattern potentially related to complex internal tumor architecture (e.g., cell-dense zones, hemorrhage, and necrosis). TTP ranged widely (6.84–28.95 s), with some cases faster than all SPN cases and others slower than most SPN cases. Parameters like mTT and FHT also exhibited extremely broad ranges, with substantial inter-case variation in their WoR values (range: 477.47–869.19). Therefore, this observation remains preliminary and descriptive. It cannot serve as a diagnostic feature for PB, and its generalizability and precise discriminatory value require further validation in large-scale studies.

### 3.4. Visualization of Representative Cases

**Figure 4:**
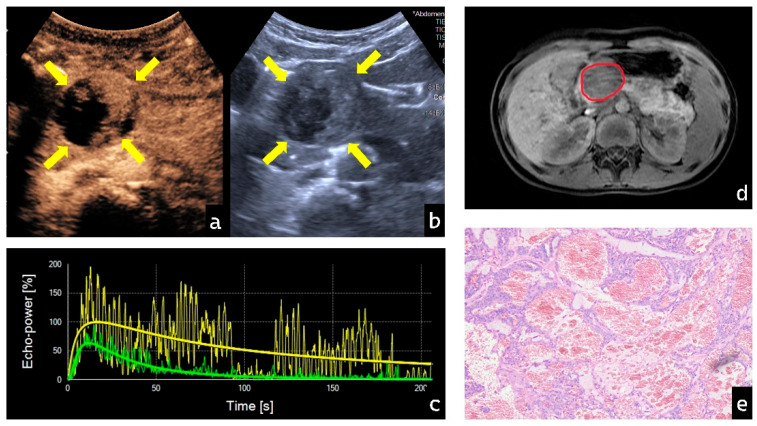
Typical multimodal imaging and pathological features of SPN female patient, aged 8 years and 11 months (**a**,**b**): Contrast-enhanced ultrasound reveals cystic-solid components within the tumor with annular enhancement of the capsule. Yellow arrow: Area of lesion (**c**) Time–intensity curve (TIC) showing green for lesion tissue and yellow for normal tissue. (**d**) MRI demonstrates a space-occupying lesion in the pancreatic head. Red circle: Area of lesion (**e**) Histopathology of the resected tumor specimen shows pseudopapillary arrangement of tumor cells surrounding blood vessels. (Cell nuclei stained purple whereas cell cytoplasm and extracellular matrix stained pink, HE × 40).

**Figure 5:**
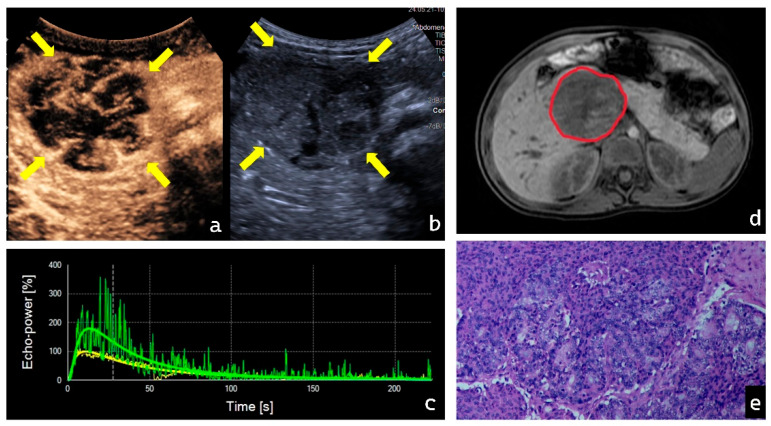
Typical multimodal imaging and pathological features of PB in a female patient, aged 1 year 11 months. (**a**,**b**) Contrast-enhanced ultrasound reveals coarse, randomly distributed trophoblastic vessels within the tumor. Yellow arrow: Area of lesion (**c**) Time–intensity curve (TIC) showing green for lesion tissue and yellow for normal tissue. (**d**) MRI demonstrates a space-occupying lesion in the pancreatic head. Red circle: Area of lesion (**e**) Histopathology of the resected tumor specimen reveals round tumor cells arranged in nests, glandular, and tubular patterns, predominantly with squamous cell nests. (Cell nuclei stained purple whereas cell cytoplasm and extracellular matrix stained pink, HE × 40).

Representative SPN and PB cases were selected to comprehensively illustrate the aforementioned qualitative and quantitative findings. Their grayscale ultrasound, contrast-enhanced ultrasound, MRI, time–intensity curve (TIC), and pathological images were integrated into a single view (Figure 4 and Figure 5).

## 4. Discussion

This study provides a systematic description of the qualitative and quantitative CEUS features of two relatively rare pediatric pancreatic tumors—spinal pancreatic neoplasms (SPN) and pancreatic blastomas (PB)—based on preliminary observations of 19 SPN and 3 PB cases. The most critical finding is that PB exhibits distinct CEUS patterns from SPN, primarily manifested as a qualitative “disorganized, coarse vascular pattern” and a quantitative “high perfusion–rapid washout” trend. The SPN group exhibited heterogeneous enhancement patterns dominated by Patterns III and II, closely matching its classic pathological architecture of intermingled solid, pseudopapillary, cystic, and hemorrhagic zones [16,17,18]. The compressed tumor stroma forms a fibrous capsule, whose rich vascular network is visualized during early CEUS enhancement. Concurrently, the fibrovascular axis is surrounded by tumor cells, with some potentially undergoing liquefactive necrosis to form cystic areas. The capsule appears more clearly delineated compared to the non-enhanced necrotic regions [19]. This characteristic of “slow, gradual, and uneven filling” constitutes its distinctive CEUS appearance, which can be used to differentiate it from other pancreatic tumors.

In contrast, all PB cases (3/3) in this study exhibited Pattern V. During the early contrast phase, PB lesions demonstrated rapid, high enhancement accompanied by chaotic internal feeding vessels, as shown in Figure 5. This directly reflects the rich and immature tumor vasculature characteristic of malignant embryonal tumors [20]. In the late phase, rapid contrast washout may indicate vascular abnormalities such as arteriovenous fistulas or increased vascular permeability, leading to swift loss of contrast microbubbles. This pronounced and rapid hemodynamic change provides an intuitive radiographic manifestation of PB’s aggressive biological behavior and serves as a key distinguishing feature from the relatively indolent SPN. In our detailed analysis of PB, we observed a seemingly contradictory yet informative phenomenon: despite all PB lesions exhibiting numerically high WoR values—indicating rapid contrast agent departure from the vascular bed—their overall mTT values remained prolonged. From the perspective of classical hemodynamic models, these two parameters appear contradictory. Based on the literature and research data, we propose a possible explanation: as a malignant embryonal tumor, PB exhibits extremely immature neovascularization, potentially featuring extensive new capillary networks [21]. This provides a “fast track” for contrast agent washout, leading to elevated WoR values. Simultaneously, dense cellular clusters within the tumor [16], high interstitial pressure, and areas of necrosis or hemorrhage create severe microcirculatory obstruction, forming low-flow zones where microbubbles experience significantly prolonged retention. Under the combined effects of these two mechanisms, the lesion exhibits a dual pattern: a portion of the contrast agent is rapidly washed out through arteriovenous fistulas, while a substantial fraction remains trapped in low-flow turbulent zones. Ultimately, this retained contrast agent “lengthens” the overall mean transit time, elevating mTT. While this hypothesis aligns with our descriptive data, it must be emphasized that its validation relies entirely on future, more detailed pathologic–imaging correlation studies of PB lesions in larger cohorts.

## 5. Limitations

EUS-guided histological examination remains the gold standard for preoperative diagnosis [22] and is established as the most valuable tool for obtaining high-quality tissue samples in these patients [23]. Complementing this, the unique microvascular perfusion information provided by CEUS in this study offers preliminary imaging evidence for noninvasive preoperative differentiation between SPN and PB. Nevertheless, these preliminary findings must be interpreted with extreme caution. This study has several important limitations: First and foremost, the extremely small sample size for PB (*n* = 3) limits the generalizability of any findings and precludes formal statistical comparisons and diagnostic performance evaluation. All conclusions should be regarded as hypothesis generation rather than definitive evidence. Second, retrospective studies may introduce selection bias. Third, this study did not perform a comparative analysis correlating CEUS features with MRI, thus failing to directly demonstrate the incremental value of CEUS over the current best imaging modality. In clinical practice, MRI remains the cornerstone for evaluating pancreatic tumors, while CEUS’s value lies in its ability to provide real-time, continuous observation of tumor microvascular perfusion, supplementing MRI with hemodynamic information. Future studies integrating CEUS and MRI into a combined diagnostic model represent a promising direction. Finally, while interobserver agreement reached “substantial agreement” (Kappa = 0.70), there remains room for improvement, suggesting that the criteria for defining certain qualitative patterns (e.g., Patterns II, III, and IV) require further refinement in future studies.

## 6. Conclusions

In summary, this preliminary study suggests that pediatric SPN and PB may exhibit distinct characteristics on CEUS. PB tends to present with rapid, high enhancement accompanied by chaotic nourishing vessels and rapid washout, whereas SPN more commonly demonstrates a moderate, slow enhancement pattern, often associated with features related to cystic components. These differences align with the distinct pathobiological characteristics of the two tumors.

Despite the strict limitations of a small sample size, the CEUS feature map outlined in this study proposes new, verifiable imaging propositions for differentiating these two rare tumors. Future research should focus on establishing multicenter collaborations to prospectively collect larger PB case series. Integrating CEUS with multiparametric MRI analysis aims to develop more reliable, quantitative diagnostic models, ultimately enabling precise, noninvasive preoperative assessment of pediatric pancreatic tumors.

## Figures and Tables

**Table 1:** Explanation of TIC Parameters.

Parameter	Significance
**IMAX**	Peak Intensity
**TTP**	Time to Peak
**RT**	Rise Time (from 10% to 90% of peak intensity)
**Rs50**	50% Rise Slope (tangent slope at 50% intensity point)
**Rs10–90**	10–90% Rise Slope (average slope between 10% and 90% intensity)
**FT**	Fall Time (from peak to baseline return)
**Fs50**	50% Fall Slope (tangent slope at 50% intensity point)
**FHT**	Fall Half Time (from peak to 50% intensity)
**mTT**	Mean Transit Time
**AUC**	Area Under Curve
**WiAUC**	Wash-in Area Under Curve (perfusion phase AUC)
**WoAUC**	Wash-out Area Under Curve (wash-out phase AUC)
**WiR**	Wash-in Rate (maximum rising slope)
**WoR**	Wash-out Rate (maximum falling slope)

**Table 2:** Baseline characteristics of patients and lesions.

Featrure	Total	SPN Group (*n* = 17)	PB Group (*n* = 3)	*p*
**age (year)**	141.42 (8.84, 14.45)	13.51 (9.87, 14.54)	2.94 (1.97, 7.39)	0.006
**Gender (Female) (%)**	18 (81.82%)	16 (84.21%)	2 (66.67%)	0.470
**Maximum tumor diameter (cm)**	5.10 (4.05, 8.25)	4.70 (3.90, 7.80)	12.7 (5.90, 7.60)	0.094
**Ki-67 (%)**	5.00 (3.00, 10.63)	5.00 (3.00, 7.00)	30.00 (30.00, 60.00)	0.006

Note: Continuous variables are expressed as median (interquartile range)/(range), while categorical variables are presented as sample size (%). Comparisons between the SPN and PB groups used the Mann–Whitney U test for continuous variables and Fisher’s exact test for categorical variables. Given the small sample size in the PB group (*n* = 3), statistical power was insufficient. *p*-values here are for reference only and should be interpreted in conjunction with descriptive statistics.

**Table 3:** Qualitative analysis features of angiography.

Feature	SPN Group	PB Group
**Pattern**		
**I**	2	0
**II**	6	0
**III**	8	0
**IV**	3	0
**V**	0	3

**Table 4:** Description of CEUS quantitative parameters for two tumor groups.

Parameter	SPN Group (*n* = 19)	PB Group (*n* = 3)
**IMAX(%)**	60.56 (36.82, 85.75)	179.84 (107.79, 192.29)
**TTP(s)**	16.01 (11.58, 20.19)	13.07 (6.84, 28.95)
**RT(s)**	15.58 (10.85, 19.85)	11.95 (5.54, 28.04)
**Rs50**	8.58 (4.12, 10.72)	26.45 (7.19, 45.78)
**Rs10–90**	6.61 (3.61, 8.31)	21.22 (6.01, 44.85)
**FT(s)**	82.68 (56.12, 131.72)	60.99 (17.85, 232.48)
**Fs50**	−0.49 (‘−1.03, −0.03)	−2.69 (‘−10.51, ’−0.40)
**FHT(s)**	39.62 (27.22, 57.72)	27.56 (8.70, 97.74)
**mTT(s)**	100.80 (50.18, 204.63)	52.64 (14.15, 305.84)
**AUC**	35,994.96 (16,735.28, 61,654.87)	91,148.06 (27,288.32, 133,900.56)
**WiAUC**	342.98 (168.44, 538.63)	1975.66 (862.24, 2084.62)
**WoAUC**	22,751.87 (9704.72, 37,536.03)	53,011.99 (13,813.84, 111,001.34)
**WiR**	24.03 (12.46, 46.67)	165.33 (30.75, 376.29)
**WoR**	275.38 (152.49, 378.68)	773.88 (477.47, 869.19)

Note: This table provides a descriptive presentation of quantitative measurements for both groups. Given the insufficient number of cases in the PB group, no intergroup statistical comparisons were performed.

## Data Availability

The raw data supporting the conclusions of this article will be made available by the authors on request.

## Supplementary Materials

The following supporting information can be downloaded at https://www.mdpi.com/article/10.3390/diagnostics16030474/s1, Table S1: Consistency analysis among observers: Intraclass correlation coefficient (ICC) of quantitative parameters of CEUS.
