# Supplementary material for: A Preliminary Study on Contrast Enhanced Ultrasound Characteristics of Solid Pseudopapillary Neoplasms and Pancreatoblastoma in Children
Source: Diagnostics (Basel). 2026 Feb 3;16(3):474. doi: 10.3390/diagnostics16030474 (PMC12896552; doi:10.3390/diagnostics16030474)
Supplement: Supplementary file 1 [file diagnostics-16-00474-s001.zip › diagnostics-4133850-supplementary.pdf]

Preliminary Study on Ultrasound Contrast Features of Solid Pseudopapillary neoplasms and Pancreatoblastoma in Children

Table S1: Consistency analysis among observers: Intraclass correlation coefficient (ICC) of quan-titative parameters of CEUS

Parameter	ICC(Single Measures)	95% CI	Agreement Evaluation
**IMAX**	0.88	0.73-0.95	Good
**TTP**	0.94	0.85-0.97	Excellent
**RT**	0.86	0.70-0.94	Good
**Rs50**	0.92	0.82-0.97	Excellent
**Rs10-90**	0.92	0.81-0.96	Excellent
**FT**	0.69	0.39-0.86	Moderate
**Fs50**	0.86	0.69-0.94	Good
**FHT**	0.71	0.42-0.87	Moderate
**mTT**	0.44	0.05-0.72	Moderate
**AUC**	0.90	0.77-0.96	Excellent
**WiAUC**	0.76	0.51-0.89	Good
**WoAUC**	0.84	0.63-0.93	Good
**WiR**	0.65	0.33-0.84	Moderate
**WoR**	0.81	0.59-0.92	Good

Note: The agreement evaluation is based on the criteria by Koo & Li: ICC < 0.50 (Poor), 0.50–0.75 (Moderate), 0.75–0.90 (Good), > 0.90 (Excellent).
